# The MCP-3/Ccr3 axis contributes to increased bone mass by affecting osteoblast and osteoclast differentiation

**DOI:** 10.1038/s12276-024-01344-6

**Published:** 2024-11-01

**Authors:** Jung Ha Kim, Kabsun Kim, Inyoung Kim, Semun Seong, Xiangguo Che, Je-Yong Choi, Jeong-Tae Koh, Nacksung Kim

**Affiliations:** 1 Department of Pharmacology, Chonnam National University Medical School, Gwangju, Republic of Korea; 2 Hard-Tissue Biointerface Research Center, School of Dentistry, Chonnam National University, Gwangju, Republic of Korea; 3 Korea Mouse Phenotyping Center (KMPC), Department of Biochemistry and Cell Biology, Cell and Matrix Research Institute, School of Medicine, Kyungpook National University, Daegu, Republic of Korea; 4 Department of Pharmacology and Dental Therapeutics, School of Dentistry, Chonnam National University, Gwangju, Republic of Korea

**Keywords:** Molecular biology, Cell signalling

## Abstract

Several CC subfamily chemokines have been reported to regulate bone metabolism by affecting osteoblast or osteoclast differentiation. However, the role of monocyte chemotactic protein 3 (MCP-3), a CC chemokine, in bone remodeling is not well understood. Here, we show that MCP-3 regulates bone remodeling by promoting osteoblast differentiation and inhibiting osteoclast differentiation. In a Ccr3-dependent manner, MCP-3 promoted osteoblast differentiation by stimulating p38 phosphorylation and suppressed osteoclast differentiation by upregulating interferon beta. MCP-3 increased bone morphogenetic protein 2-induced ectopic bone formation, and mice with MCP-3-overexpressing osteoblast precursor cells presented increased bone mass. Moreover, MCP-3 exhibited therapeutic effects by abrogating receptor activator of nuclear factor kappa-B ligand-induced bone loss. Therefore, MCP-3 has therapeutic potential for diseases involving bone loss due to its positive role in osteoblast differentiation and negative role in osteoclast differentiation.

## MCP-3 Enhances Bone Formation and Reduces Bone Loss

Our bones are always changing, needing a balance between bone creation and bone breakdown. Researchers investigates how a specific protein, MCP-3, affects this balance. They found that MCP-3 promotes the creation of osteoblasts and stops the creation of osteoclasts. The study involved both in vitro and in vivo methods, looking at how changing MCP-3 levels impacts bone change processes. Researchers found that increasing MCP-3 levels in bone-making cells resulted in stronger bones in mice, suggesting MCP-3 is key in promoting bone creation and stopping excessive bone loss. This was partly due to MCP-3’s interaction with specific cell receptors and signaling pathways that affect bone cell activity. This research paves the way for future studies on MCP-3 as a target for bone-strengthening therapies, potentially leading to new treatments for diseases that weaken bones. This summary was initially drafted using artificial intelligence, then revised and fact-checked by the author.

## Introduction

Maintaining normal skeletal function and preserving bone mass require a tightly balanced bone remodeling process, whereby bone-forming osteoblasts and bone-resorbing osteoclasts function together to sequentially reconstruct the skeleton^1^. The cytokines macrophage colony-stimulating factor (M-CSF) and receptor activator of nuclear factor kappa-B ligand (RANKL) are essential for osteoclastogenesis, whereas bone morphogenetic protein (BMP) cytokines are crucial for osteoblastogenesis^2,3^. Moreover, some immune cell cytokines, including interleukin-1, interleukin-6, tumor necrosis factor-α/β, interferon-γ, and some chemokines and their receptors, reportedly modulate bone cell differentiation and function^4–11^.

Although chemokines were initially identified because of their capacity to attract leukocytes to inflammatory sites, these molecules were later shown to have protective effects on various cell types, including bone cells^12^. Chemokine ligand 7 (CCL7), which is also known as monocyte chemotactic protein 3 (MCP-3), was identified in an osteosarcoma supernatant and is a member of the CC chemokine subfamily^13^. Under physiological conditions, several cell types, including stromal cells, airway smooth muscle cells, and keratinocytes, express MCP-3^14^. However, MCP-3 is also expressed under pathological conditions, including by tumor cells. MCP-3 potently chemoattracts several types of leukocytes, including monocytes, eosinophils, basophils, dendritic cells, natural killer cells, and activated T lymphocytes^12,15^. Chemokine function depends on interactions with their receptors on target cells. Specifically, by binding to various receptors, including CC motif chemokine receptor (CCR)1, CCR2, CCR3, CCR5, and CCR10, MCP-3 acts on a wide range of innate and adaptive immune cells^16–18^.

In marrow cultures, CCR1 chemokines, including MCP-3, MCP-1, and CCL5, were shown to directly and strongly increase osteoclast formation via a RANKL-dependent pathway^19^. However, contentious findings have also been reported. For example, compared with wild-type conditions, *Ccl5* deficiency reportedly increased osteoclastogenesis in vitro and in vivo^4^. Furthermore, deficiency in CCR3, which binds to various ligands, including MCP-3 and CCL5, reportedly increased osteoclast size and the number of nuclei per osteoclast^20^. Because some chemokines bind to multiple receptors and because these receptors may share chemokines from the same family, the network between chemokines and their receptors is very complex^21^. Therefore, as with CCL5, MCP-3 may also influence osteoclastogenesis through non-CCR1 receptors, which warrants further investigation.

Monocytes express high levels of irisin, which was reported to stimulate bone cell signaling pathways and promote osteoblast proliferation^22^. A recent study showed that irisin promotes C2C12 myoblast proliferation by increasing MCP-3 expression^22,23^. However, it is unclear whether irisin stimulates bone cell signaling pathways by upregulating MCP-3 expression and whether MCP-3 regulates bone remodeling by influencing osteoblast differentiation and function.

Here, we investigated whether MCP-3 participates in bone remodeling by influencing osteoblastogenesis and osteoclastogenesis and sought to identify its main receptors during bone cell differentiation and function.

## Materials and methods

### Mice

All animal experiments were approved by the Chonnam National University Medical School Research Institutional Animal Care and Use Committee (IACUC-H-2021-58). All animal experiments adhered to the approved guidelines. Transgenic MCP-3 mice were generated by Macrogen, Inc. (Seoul, Korea), by fusing full-length *Mcp-3* to the mouse *Prx1* gene promoter, followed by standard pronuclear injection into C57BL/6 mice. The mice were genotyped via PCR analysis of tail snip genomic DNA with the following primers: CCTTTCTCTCTGGCTCTGATG and GCTTCAGCACAGACTTCCATG.

### Reagents

Recombinant human RANKL was purified from bacteria. Recombinant human M-CSF was a gift from Dr. Daved Fremont (Washington University, St. Louis, MO, USA). Recombinant human BMP2 protein, ascorbic acid, β-glycerophosphate, and recombinant mouse MCP-3 were purchased from Cowellmedi (Busan, Korea), Junsei Chemical (Tokyo, Japan), Sigma–Aldrich (St. Louis, MO, USA), and Cloud-Clone Corp. (Wuhan, China), respectively.

### Osteoclast differentiation

For bone marrow-derived macrophages (BMMs, osteoclast precursors), bone marrow cells, which were extracted from the femurs and tibias of Institute of Cancer Research (ICR) mice, were cultured in α-MEM supplemented with 10% fetal bovine serum and M-CSF (30 ng/ml) for three days. The BMMs were cultured further in α-MEM supplemented with 10% fetal bovine serum, M-CSF (30 ng/ml), and RANKL (20–150 ng/ml), with or without MCP-3 (5–100 ng/ml). The cultured cells were then fixed with 3.7% formalin, treated with TRAP staining solution (Sigma–Aldrich) for 10 min, imaged via a ProRes CFscan camera (Jenoptik, Jena, Germany), and analyzed via ProgRes Capture Pro software. TRAP-positive cells containing more than three nuclei were identified as osteoclasts.

### Osteoblast differentiation

Enzymatic digestion was used to isolate primary osteoblast precursor cells from neonatal mouse calvariae. After the calvariae were treated with 0.1% collagenase (Life Technologies, Carlsbad, CA) and 0.2% dispase II (Roche Diagnostics Gmbh, Mannheim, Germany) for 10 min at 37 °C, the first batch of obtained cells was discarded. Next, four sequential 10-minute enzymatic digestions were used to obtain primary osteoblast precursor cells from the calvariae. Osteoblast precursor cells were cultured in α-MEM supplemented with 10% fetal bovine serum, BMP2 (100 ng/ml), ascorbic acid (50 μg/ml), and β-glycerophosphate (10 mM), with or without MCP-3 (100 ng/ml). For analysis of ALP activity, cultured cells were lysed, followed by lysate incubation in p-nitrophenyl phosphate substrate (Sigma–Aldrich, St. Louis, MO). ALP activity was then determined by reading the absorbance at 405 nm on a spectrophotometer. Cultured cells were fixed with 3.7% formalin, followed by treatment with Alizarin Red (40 mM, pH 4.2) for 10 min and scanning of the stained culture plates on a CanoScan 9900 F system (Canon, Inc., Japan). For substrate calcification quantification, 10% acetic acid was used to extract Alizarin Red, after which its concentration was measured through absorbance readings at 405 nm.

### Western blotting

Total protein was extracted via lysis buffer (50 mM Tris-HCl [pH 8.0], 150 mM NaCl, 1 mM EDTA, 0.5% Nonidet P-40, and 1 mM PMSF) supplemented with a protease inhibitor cocktail. The protein concentration was then measured via a BCA protein assay kit (Pierce, Rockford). Equal protein amounts were then resolved via sodium dodecyl sulfate–polyacrylamide gel electrophoresis and transferred onto a polyvinylidene fluoride membrane (Millipore, Billerica, MA). The membranes were then blocked with 5% skim milk and incubated with primary antibodies against actin (Sigma–Aldrich), c-Fos (Santa Cruz Biotechnology, Santa Cruz, CA), NFATc1 (Santa Cruz Biotechnology), Cathepsin K (Santa Cruz Biotechnology), phospho-p38, p38, phospho-JNK, JNK, phospho-AKT, AKT, phospho-SMAD158, SMAD158, and IκB (all: Cell Signaling Technology, Beverly, MA). The samples were then washed and incubated with appropriate horseradish peroxidase-conjugated secondary antibodies. The signal was then developed via an enhanced chemiluminescence substrate (Millipore), followed by visualization of the results via an LAS3000 luminescent image analyzer (GE Healthcare, Piscataway, NJ).

### Real-time quantitative PCR

RNA was isolated from cultured cells via Qiazol (Qiagen GmbH, Hilden, Germany) according to the manufacturer’s protocol. Reverse transcription was performed via a QuantiNova Reverse Transcription Kit (Qiagen). Real-time quantitative PCR analyses were performed on a Rotor-Gene Q system (Qiagen). The comparative CT method was used to determine relative mRNA levels, with *Gapdh* used as the reference gene. The following primers (gene name, forward primer, reverse primer) were used: *c-fos*, ATGGGCTCTCCTGTCAACACACAG, TGGCAATCCAGTCTGCAACGCAG; *Nfatc1*, CTCGAAAGACAGCACTGGAGCAT, CGGCTGCCTTCCGTCTCATAG; *Acp5*, TCCGTGCTCGGCGAGGACCAGA, CTGGAGTGCACGATGCCAGCGACA; *Ctsk*, ACGGAGGCATTGACTCTGAGATG, GTTGTTCTTATTCCGAGCCAAGAG; *Runx2*, CCCAGCCACCTTTACCTA-CA, CAGCGTCAACACCATCATTC; *Alpl*, CAAGGATATCGACGTGATCATG, GTCAGTCAGGTTGTTCCGATTC; *Ibsp*, GGAAGAGGAGACTTCAAACGAAG, CATCCACTTCTGCTTCTTCGTTC; *Mcp-3*, GTGCCTGAACAGAAACCAACCT, CATTCCTTAGGCGTGACCATT; *Ccr1*, CCATTGTCCATGCTGTGTTT, GCAAAATTAGTCCAAGAAGGTT; *Ccr2*, TCATCCACGGCATACTATCAA, TATTCCCAAAGACCCACTCAT; *Ccr3*, TTACCTGGCCTTGTACAGCG, GGATAGCGAGGACTGCAGGA; *Ccr5*, TGCTGTGTTTGCTTTAAAAG, ATGACAAGTAGAGGCAGGAT; *Gapdh*, TGACCACAGTCCATGCCATCACTG, CAGGAGACAACCTGGTCCTCAGTG.

### Enzyme-linked immunosorbent assay

Blood was collected from euthanized mice, allowed to clot, and then centrifuged to obtain serum. Next, procollagen I NT propeptide (Novatein Biosciences, Woburn, MA), type I collagen crosslinked C telopeptide (Novatein Biosciences), and MCP-3 (Mybiosource, San Diego, CA) serum levels were measured via enzyme-linked immunosorbent assay kits according to the manufacturer’s instructions.

### Microcomputed tomography analysis

A SkyScan 1172 system (SkyScan, Kotich, Belgium) was used to scan and analyze isolated-distal femurs via the following scanning parameters: 50 kV, 201 μA, 0.5 mm aluminum filter, and a resolution of 11 μm per pixel. Images were captured at 0.7° intervals over an angular range of 180°. The raw images were reconstructed into serial cross-sections. Image reconstruction software (NRecon 1.4, SkyScan), data analysis software (CTAn, SkyScan), and three-dimensional model visualization software (Ant 2.4, SkyScan) were used for femoral morphometric parameter analysis.

### Bone histomorphometry

Nine and two days before euthanasia, the mice were intraperitoneally injected with calcein (20 mg/kg, Sigma–Aldrich). Undecalcified femurs were fixed with formalin, dehydrated, and then embedded in destabilized methyl methacrylate. Undecalcified femurs were sagittally sectioned from the distal metaphysis to the midshaft region at a thickness of 5 mm. Histomorphometric parameters were measured via the BioQuant Osteo IIImage Analysis System, Version 8.00.20 (BioQuant Image AnalysisCorp, Nashville, TN, USA), and the parameters were calculated in the same area as the bone volume. Calcein labeling was examined via confocal microscopy (Olympus, Tokyo, Japan), and histomorphometric analysis of the bone compartment was conducted via Kappa Image Base Control 2.3.5 software (KAPPA Opto-electronics, GmbH, Gleichen, Germany). For TRAP and hematoxylin and eosin staining, tibias were fixed with 4% paraformaldehyde, decalcified, dehydrated, embedded in paraffin blocks, and then longitudinally sectioned at a thickness of 4 μm. The sections were then deparaffinized with xylene, followed by staining with TRAP and hematoxylin and eosin.

### Ectopic bone formation

Four-week-old male ICR mice were anesthetized through intraperitoneal injection of 0.1% avertin (Sigma). Collagen sponges soaked with BMP2 (1 µg) + PBS or BMP2 (1 µg) + MCP-3 (2 µg, Cloud-Clone Corp., Wuhan, China) were individually implanted under the dorsal skin on both sides of anesthetized mice. After four weeks, the mice were euthanized with CO_2_, followed by bone generation analysis.

### RANKL-induced bone loss

Six-week-old, healthy male ICR mice were divided randomly into the RANKL/MCP-3, RANKL/PBS, and RANKL and MCP-3 groups. One day before RANKL injection, the RANKL/MCP-3 group received MCP-3 (0.2 mg/kg) via intraperitoneal injection. The next day, the RANKL/PBS group received RANKL (10 mg/kg) injections intraperitoneally, whereas the RANKL and MCP-3 groups received intraperitoneal RANKL and MCP-3 injections for three consecutive days. If RANKL or MCP-3 was not injected, PBS was intraperitoneally injected at the corresponding volumes. On day five, the mice were euthanized, followed by bone phenotype analysis.

### Statistical analyses

The data are presented as the means ± SDs. Statistical differences between two groups were identified via two-tailed Student’s t tests. Differences between multiple groups were identified via analysis of variance with post hoc Tukey’s HSD test. *P* < 0.05 indicated statistically significant differences.

## Results

### MCP-3 stimulates osteoblast differentiation and bone nodule formation

**Fig. 1:**
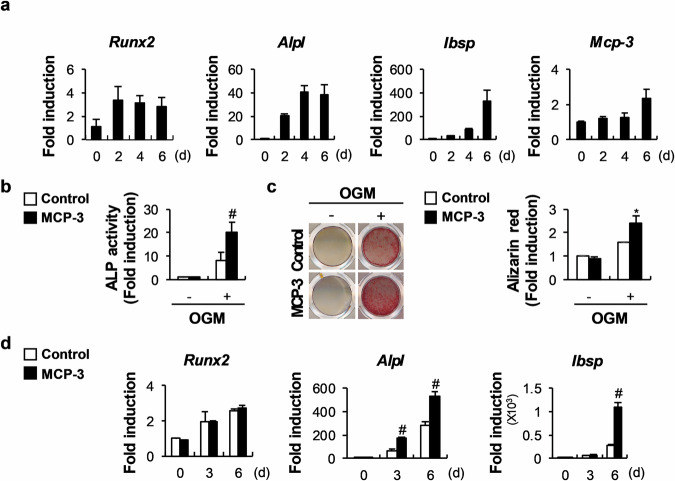
MCP-3 promotes osteoblast differentiation and function. **a** Osteoblasts were cultured in osteogenic medium (OGM). The relative mRNA levels of the indicated genes were determined via RT‒qPCR (*n* = 3). **b**–**d** Osteoblasts were cultured in OGM with or without MCP-3. **b** After culture for three days, the cells were lysed and subjected to alkaline phosphatase (ALP) activity measurement (*n* = 3). **c** After culture for six days, the cells were stained with Alizarin Red and quantified via extraction (*n* = 3). **d** The relative mRNA levels of the indicated genes were determined via RT‒qPCR (*n* = 3). The data are presented as the means ± SDs of triplicate samples. # and * indicate *p* < 0.05 and < 0.01, respectively, vs. the control.

We first examined MCP-3 expression during osteoblast differentiation and found that culturing calvarial osteoblasts in BMP2-, ascorbic acid-, and β-glycerol phosphate-containing osteogenic media elevated the mRNA levels of osteoblast markers such as Runt-related transcription factor 2 (*Runx2*), alkaline phosphatase (ALP, *Alpl*), and bone sialoprotein (BSP, *Ibsp*) as the osteoblasts differentiated (Fig. 1a). We found that osteoblasts express MCP-3 and that its mRNA levels increase slightly during osteoblastogenesis (Fig. 1a). Compared with the control, treatment with MCP-3 significantly increased ALP activity and matrix mineralization levels during osteoblastogenesis (Fig. 1b–c). Furthermore, MCP-3 increased the gene expression of osteoblast markers, such as *Alpl* and *Ibsp*, but not *Runx2* (Fig. 1d). To confirm its role in osteoblasts, we retrovirally overexpressed MCP-3 in calvarial osteoblasts and observed that, compared with the control, MCP-3 increased ALP activity and nodule formation (Supplementary Fig. 1). These results indicate that MCP-3 positively regulates osteoblast differentiation and function via autocrine and paracrine effects.

### MCP-3 mediates its effects on osteoblasts via p38 phosphorylation

**Fig. 2:**
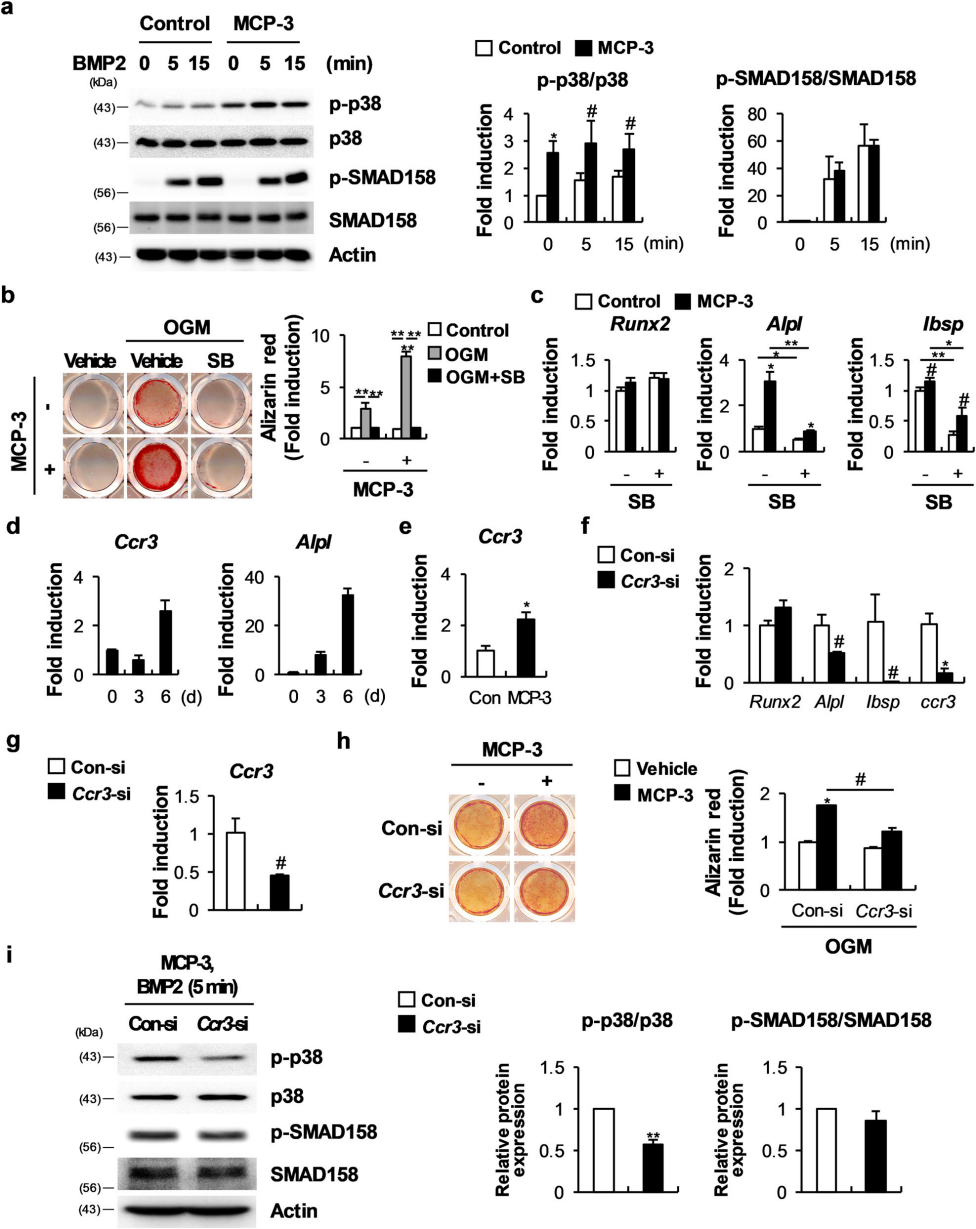
In osteoblasts, MCP-3 regulates nodule formation by increasing p38 phosphorylation in a Ccr3-dependent manner. **a** Serum-starved osteoblasts pretreated with or without MCP-3 for 60 min were stimulated with bone morphogenetic protein 2 (BMP2) for the indicated times, followed by western blot analysis of the indicated proteins (*n* = 3). **b**, **c** Osteoblasts were cultured in OGM with or without MCP-3 and SB203580. **b** Cultured cells were stained with Alizarin Red and quantified via extraction (*n* = 3). **c** The relative mRNA levels of the indicated genes were determined via RT‒qPCR (*n* = 3). **d** Osteoblasts were cultured in OGM, and the relative mRNA levels of the indicated genes were determined via RT‒qPCR (*n* = 3). **e** Osteoblasts were cultured in the presence or absence of MCP-3, and the relative Ccr3 mRNA level was determined via RT‒qPCR (*n* = 3). **f** Osteoblasts were transfected with Con-siRNA or *Ccr3*-siRNA and then cultured in OGM. The relative mRNA levels of the indicated genes were determined via RT‒qPCR (*n* = 3). **g**, **h** Osteoblasts were transfected with Con-siRNA or *Ccr3*-siRNA and cultured with OGM in the presence or absence of MCP-3. **g** Relative Ccr3 mRNA levels were determined via RT‒qPCR (*n* = 3). **h** Cultured cells were stained with Alizarin Red and quantified via extraction (*n* = 3). **i** After transfection with Con-siRNA or *Ccr3*-siRNA, osteoblasts were serum-starved, pretreated with MCP-3, stimulated with BMP2, and then subjected to western blot analysis of the indicated proteins (*n* = 3). The data are presented as the means ± SDs of triplicate samples. #, *, and ** indicate *p* < 0.05, < 0.01, and < 0.001, respectively, vs. the control.

Analysis of the signaling pathways that MCP-3 might regulate revealed that it promotes the phosphorylation of p38 but not SMAD158 (Fig. 2a)^24^. Moreover, the MCP-3-induced increase in nodule formation was markedly inhibited by SB203580, a pharmacological p38 inhibitor (Fig. 2b). Additionally, the mRNA levels of osteoblast markers such as *Alpl* and *Ibsp* were regulated by MCP-3 and SB203580, similar to nodule formation (Fig. 2c). However, compared with those in the control, in the presence of MCP-3, the *Alpl* and *Ibsp* expression levels remained slightly elevated upon treatment with SB203580 (Fig. 2c). These data indicate that, in part, MCP-3 promotes osteoblast differentiation and function by increasing p38 phosphorylation.

### Ccr3 is a functional MCP-3 receptor during osteoblastogenesis

To identify functional MCP-3 receptors during osteoblast differentiation, we first examined the expression of the CC motif chemokine receptors Ccr1, Ccr2, Ccr3, and Ccr5 and confirmed that only Ccr3 expression was slightly elevated during osteoblast differentiation (Fig. 2d). Interestingly, treating osteoblast progenitor cells with MCP-3 significantly increased *Ccr3* expression (Fig. 2e), whereas compared with control siRNA, siRNA targeting *Ccr3* significantly suppressed *Alpl* and *Ibsp* expression (Fig. 2f). Moreover, *Ccr3* downregulation inhibited nodule formation and p38 phosphorylation, which were increased by MCP-3 (Fig. 2g–i). These observations indicate that MCP-3 modulates osteoblast differentiation and nodule formation via Ccr3.

### MCP-3 attenuates RANKL-induced osteoclastogenesis

**Fig. 3:**
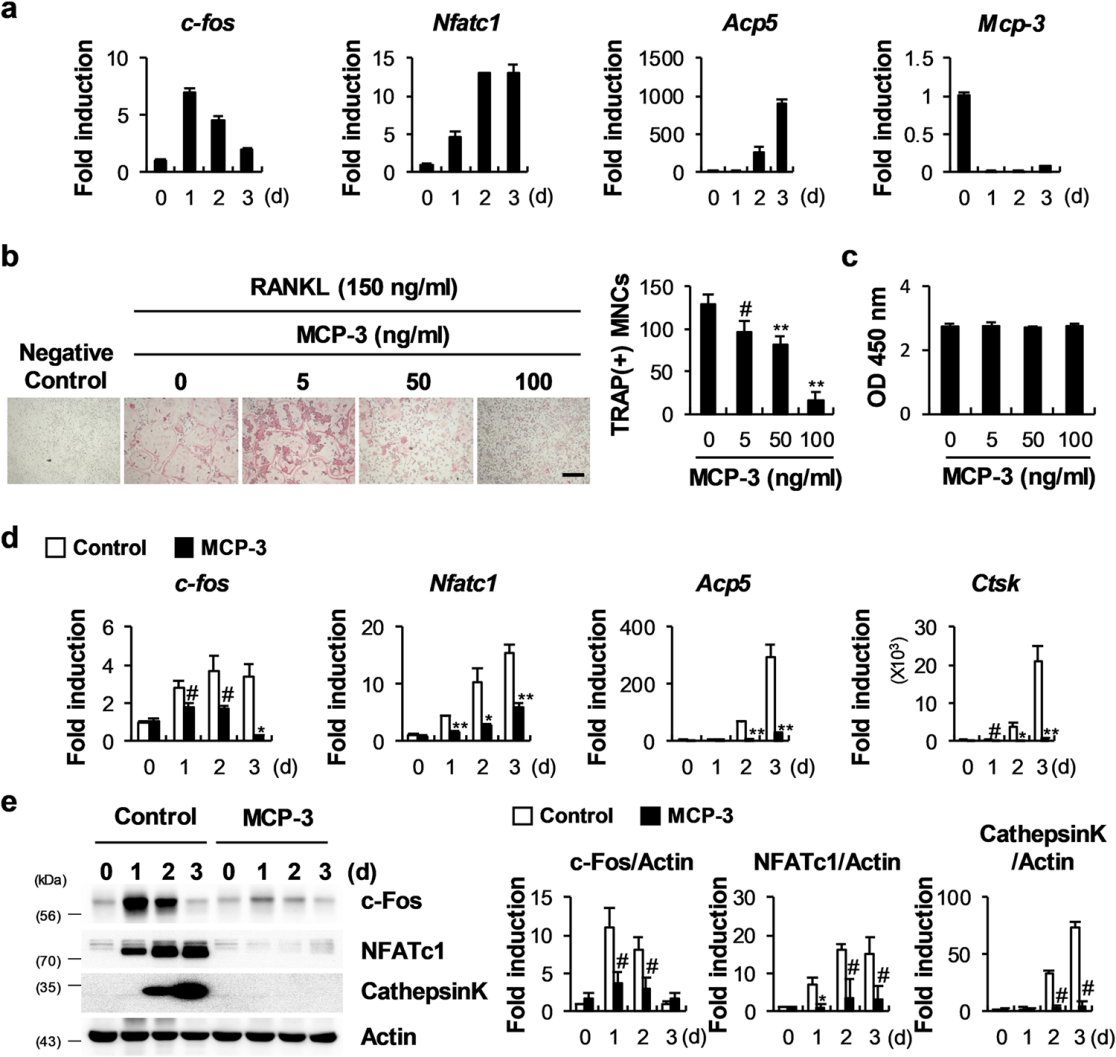
MCP-3 inhibits osteoclast differentiation. **a** Bone marrow-derived macrophages (BMMs) were differentiated with macrophage colony-stimulating factor (M-CSF) and receptor activator of NF-κB ligand (RANKL). The relative mRNA levels of the indicated genes were determined via RT‒qPCR (*n* = 3). **b** BMMs were differentiated with M-CSF and RANKL and treated with the indicated MCP-3 concentrations during osteoclast differentiation. The cultured cells were stained with tartrate-resistant acid phosphatase (TRAP), and TRAP-positive cells were counted (*n* = 3; scale bar: 200 µm). **c** BMMs were cultured with M-CSF and treated with the indicated MCP-3 concentrations, followed by an MTT assay after three days. **d**, **e** BMMs were differentiated with M-CSF and RANKL with or without MCP-3. **d** The relative mRNA levels of the indicated genes were determined via RT‒qPCR (*n* = 3). **e** The indicated proteins were assayed via western blot analysis (*n* = 3). The data are presented as the means ± SDs of triplicate samples. #, *, and ** indicate *p* < 0.05, < 0.01, and < 0.001, respectively, vs. the control.

Next, to confirm its role in osteoclast differentiation, we investigated MCP-3 expression during RANKL-induced osteoclast differentiation. This analysis revealed high MCP-3 expression in osteoclast precursor cells, which was strongly inhibited by RANKL (Fig. 3a). Moreover, MCP-3 expression inhibited by RANKL was restored by treatment with SB203580, indicating that RANKL inhibits MCP-3 expression via p38 phosphorylation (Supplementary Fig. 2). Treatment with MCP-3 significantly inhibited osteoclast formation in a dose-dependent manner but did not affect their proliferation (Fig. 3b–c). Furthermore, MCP-3 inhibited the expression of osteoclast differentiation-related genes, including c-Fos, NFATc1, TRAP (*Acp5*), and cathepsin K (*Ctsk*), at the mRNA and protein levels (Fig. 3d–e). Next, to confirm its role in osteoclastogenesis, we retrovirally overexpressed MCP-3 in osteoclast precursor cells and observed that, compared with the control conditions, MCP-3 overexpression inhibited RANKL-induced osteoclast formation (Supplementary Fig. 3). Together, these results indicate that MCP-3 negatively regulates osteoclast differentiation via autocrine and paracrine effects.

### MCP-3 inhibits osteoclast differentiation via Ccr3-dependent and Ccr3-independent pathways

**Fig. 4:**
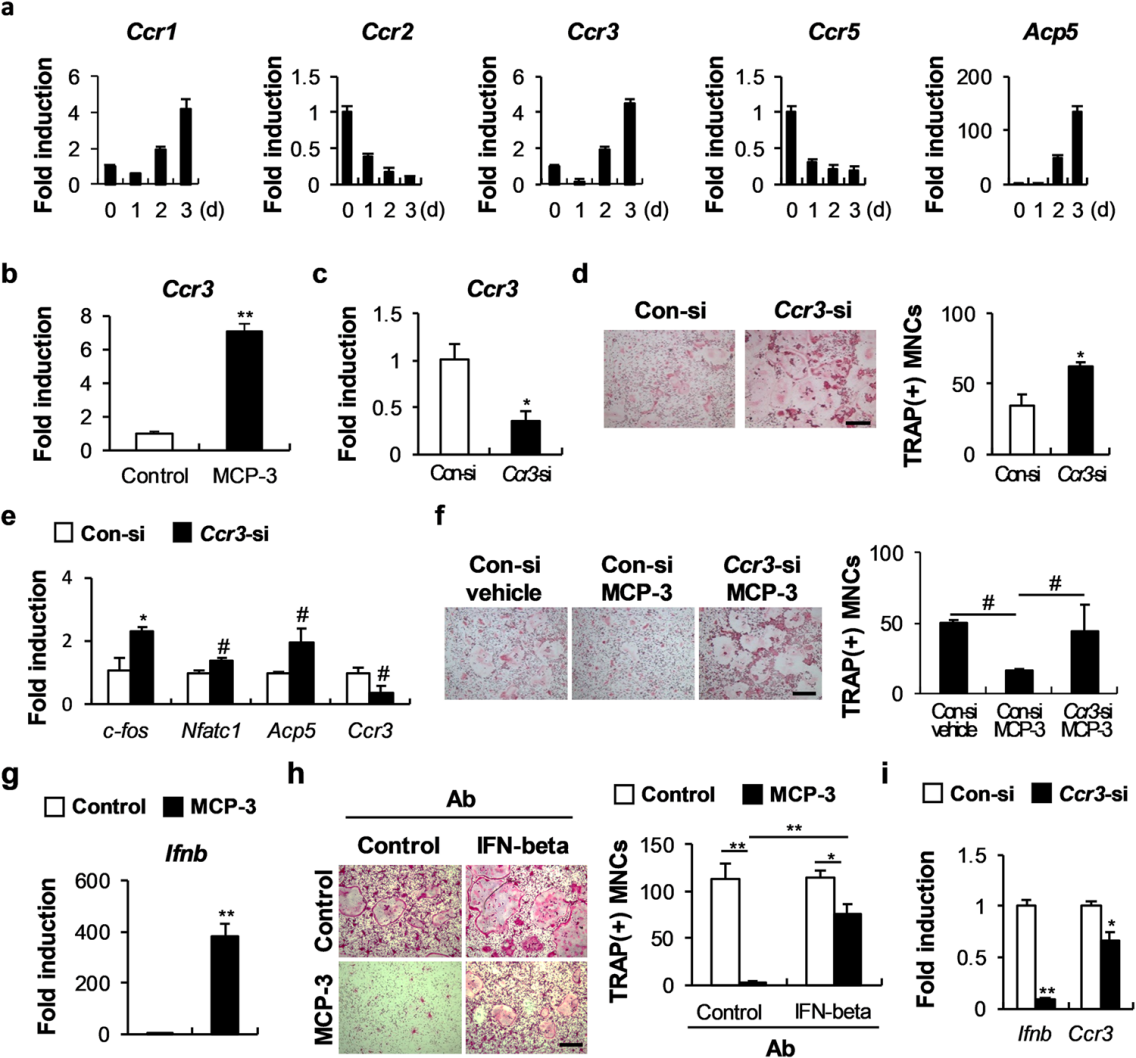
MCP-3 inhibits osteoclast differentiation via Ccr3-dependent interferon beta (IFNβ) upregulation. **a** BMMs were differentiated with M-CSF and RANKL, and the relative mRNA levels of the indicated genes were determined via RT‒qPCR (*n* = 3). **b** BMMs were cultured with M-CSF with or without MCP-3. The relative *Ccr3* mRNA level was determined via RT‒qPCR (*n* = 3). **c** Con-siRNA- or *Ccr3*-siRNA-transfected BMMs were cultured with M-CSF, and the relative *Ccr3* mRNA level was determined via RT‒qPCR (*n* = 3). **d**, **e** BMMs were transfected with Con-siRNA or Ccr3-siRNA and then cultured with M-CSF and RANKL. **d** Cultured cells were stained with TRAP, and TRAP-positive cells were counted (*n* = 3; scale bar: 200 µm). **e** The relative mRNA levels of the indicated genes were determined via RT‒qPCR (*n* = 3). **f** BMMs were transfected with Con-siRNA or *Ccr3*-siRNA and cultured with M-CSF and RANKL, with or without MCP-3. The cultured cells were stained with TRAP, and TRAP-positive cells were counted (*n* = 3; scale bar: 200 µm). **g** BMMs were cultured with M-CSF with or without MCP-3, and the relative *Ifnb* mRNA level was determined via RT‒qPCR (*n* = 3). **h** BMMs were cultured with M-CSF and RANKL, with or without MCP-3 and an IFNβ-blocking antibody. The cultured cells were stained with TRAP, and TRAP-positive cells were counted (*n* = 3; scale bar: 200 µm). **i** BMMs were transfected with Con-siRNA or *Ccr3*-siRNA and then cultured with M-CSF and RANKL. The relative mRNA levels of the indicated genes were determined via RT‒qPCR (*n* = 3). The data are presented as the means ± SDs of triplicate samples. #, *, and ** indicate *p* < 0.05, < 0.01, and < 0.001, respectively, vs. the control.

Analysis of whether Ccr3 acts as an MCP-3 functional receptor in osteoclasts revealed that Ccr3, Ccr1, Ccr2, and Ccr5 were expressed during RANKL-induced osteoclast differentiation and that Ccr3 expression increased gradually with osteoclast differentiation (Fig. 4a). Interestingly, as in osteoblasts, treating osteoclast precursor cells with MCP-3 strongly increased *Ccr3* expression (Fig. 4b). Moreover, *Ccr3* downregulation in osteoclast precursor cells not only increased RANKL-induced osteoclast differentiation but also significantly restored MCP-3-suppressed osteoclast formation (Fig. 4c–f). Our investigation of which signaling pathways for osteoclast differentiation might be regulated by MCP-3^25^ revealed that RANKL induced the phosphorylation of p38, JNK, and Akt, as well as IκB degradation. Moreover, MCP-3 significantly inhibited JNK phosphorylation and IκB degradation (Supplementary Fig. 4a). However, unexpectedly, *Ccr3* downregulation did not affect JNK phosphorylation or IκB degradation, which are regulated by MCP-3 (Supplementary Fig. 4b). The expression of c-Fos, a key gene that is induced early in osteoclast differentiation, was suppressed by MCP-3 (Fig. 3). Because interferon beta (IFNβ) can inhibit osteoclast differentiation by suppressing c-Fos expression, we examined whether MCP-3 regulates IFNβ expression in osteoclast precursor cells^26^. Treating osteoclast precursor cells with MCP-3 strongly increased *Ifnb* expression, and IFNβ neutralization significantly blocked the MCP-3-mediated inhibition of RANKL-induced osteoclast differentiation (Fig. 4g–h). Moreover, during osteoclastogenesis, *Ccr3* downregulation inhibited *Ifnb* expression (Fig. 4i). Together, these results indicate that MCP-3 either suppresses RANKL-induced JNK phosphorylation and IκB degradation independently of Ccr3 or induces IFNβ in a Ccr3-dependent manner, thereby inhibiting osteoclast differentiation.

### MCP-3 contributes to increased bone mass in vivo

**Fig. 5:**
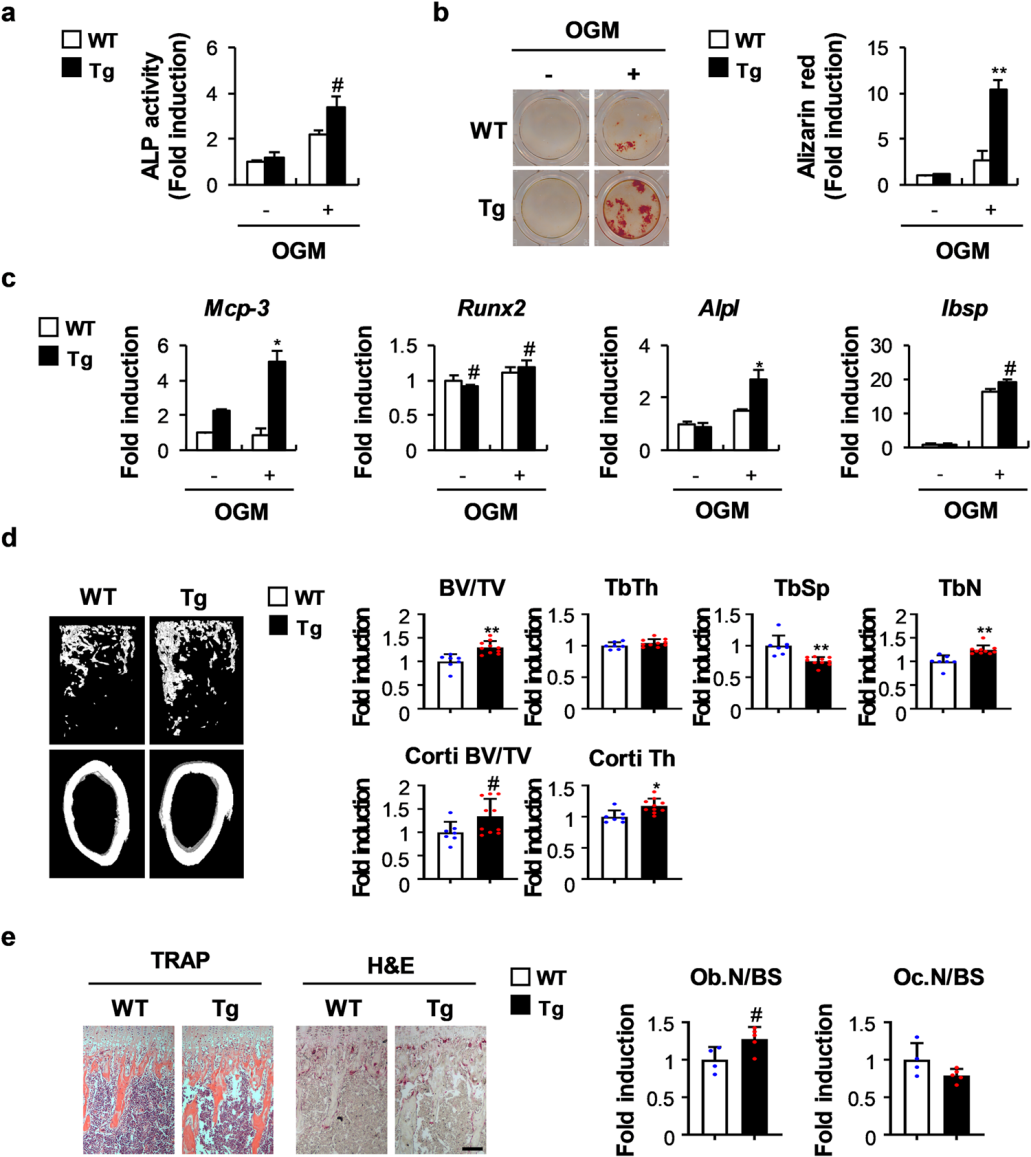
Transgenic mice overexpressing osteoblast-specific MCP-3 presented increased long-bone mass. **a**–**c** Bone marrow stromal cells from MCP-3 transgenic mice or their wild-type littermates were cultured with or without OGM. **a** ALP activity was measured (*n* = 3). **b** Cultured cells were stained with Alizarin Red and quantified via extraction (*n* = 3). **c** The relative mRNA levels of the indicated genes were determined via RT‒qPCR (*n* = 3). **d**, **e** Microcomputed tomography (µCT) and histological analyses of long bones from MCP-3 transgenic mice or their wild-type littermates. **d** Representative µCT 3D images of femurs from MCP-3 transgenic mice or their wild-type littermates. The bone volume/tissue volume (BV/TV), trabecular thickness (Tb.Th), trabecular separation (Tb.Sp), trabecular number (Tb.N), cortical bone volume/tissue volume (Corti BV/TV), and cortical thickness (Corti Th) were determined via µCT (*n* = 7 or 10). **e** Hematoxylin and eosin- and TRAP-stained images of tibiae from MCP-3 transgenic mice or their wild-type littermates. Osteoblasts and osteoclasts were quantified via histological analyses (*n* = 4 or 5). The data are presented as the means ± SDs. #, *, and ** indicate *p* < 0.05, < 0.01, and < 0.001, respectively, vs. the control.

**Fig. 6:**
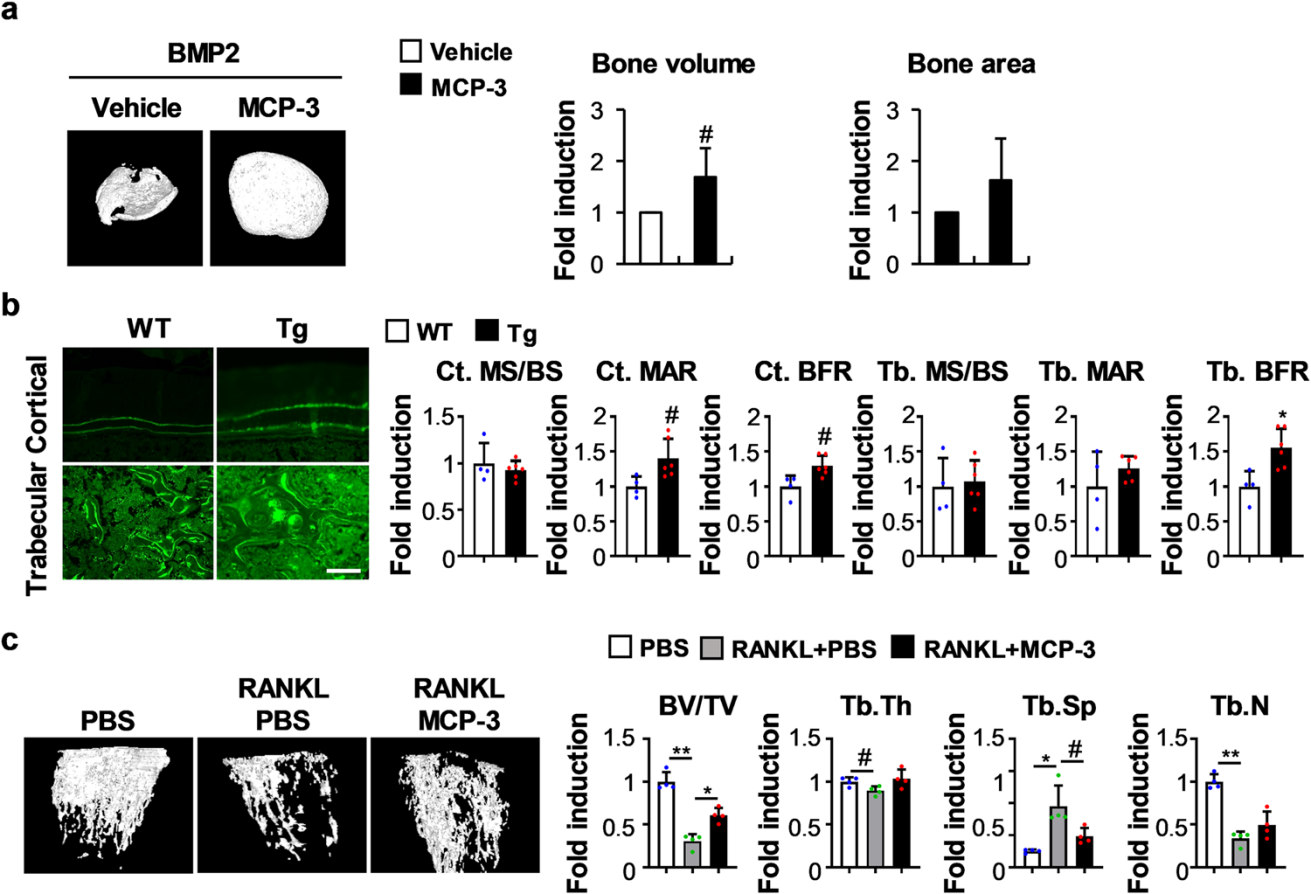
MCP-3 increases bone formation in vivo. **a** Collagen sponges soaked with BMP2, with or without MCP-3, were subcutaneously implanted on top of the dorsal back. Ectopic bones were biopsied and subjected to µCT analyses. Representative µCT 3D ectopic bone images are shown. The bone volume and bone area were determined via µCT (*n* = 5). **b** Ten and two days before euthanasia, MCP-3 transgenic mice and their wild-type littermates were intraperitoneally injected with calcein green. Representative calcein double-labeled images are shown. The cortical bone mineralizing surface/bone surface (Ct. MS/BS), cortical bone mineral apposition rate (Ct. MAR), cortical bone formation rate (Ct. BFR), trabecular mineralizing surface/bone surface (Tb. MS/BS), trabecular mineral apposition rate (Tb. MAR), and trabecular bone formation rate (Tb. BFR) were assessed (*n* = 4 or 6, scale bar: 50 µm). **c** Mice were intraperitoneally injected with PBS or MCP-3 one day before RANKL injection. RANKL and PBS or RANKL and MCP-3 were injected daily (simultaneously) for three days. Representative 3D femur images are shown. The bone volume/tissue volume (BV/TV), trabecular thickness (Tb.Th), trabecular separation (Tb.Sp), and trabecular number (Tb.N) were assessed via µCT (*n* = 4). The data are presented as the means ± SDs. #, *, and ** indicate *p* < 0.05, < 0.01, and < 0.001, respectively, vs. the control.

To investigate the in vivo effects of MCP-3 on bone mass, we generated a transgenic mouse model overexpressing MCP-3 in osteoblast precursor cells. A comparison of MCP-3 expression in cells from wild-type mice and transgenic MCP-3 mice revealed no differences in BMMs (data not shown). However, MCP-3 expression was greater in the bone marrow stromal cells (BMSCs) from the transgenic MCP-3 mice (Fig. 5). Moreover, compared with wild-type mouse BMSCs, those from transgenic MCP-3 mice presented increased ALP activity and nodule formation (Fig. 5a–b). Additionally, compared with wild-type mouse BMSCs, those from transgenic MCP-3 mice expressed higher levels of osteoblast genes, such as *Alpl* and *Ibsp* (Fig. 5c). Microcomputed tomography revealed that, compared with wild-type mice, transgenic MCP-3 mice presented markedly greater trabecular bone volume, trabecular number, cortical bone volume, and cortical thickness, as well as significantly lower trabecular separation (Fig. 5d). Moreover, bone histological analyses revealed that, compared with wild-type mice, transgenic MCP-3 mice presented significantly greater osteoblast numbers and slightly (but not significantly) lower osteoclast numbers (Fig. 5e). These results suggest that transgenic mice that specifically overexpress MCP-3 in osteoblast precursor cells have increased bone mass because of the promotion of osteoblastic bone formation. The benefits of the MCP-3-mediated increase in in vivo bone mass were further confirmed in three mouse models. Ectopic BMP2-mediated bone formation was increased by MCP-3, and calcein double-staining revealed that, compared with wild-type mice, transgenic-MCP-3 mice had more newly formed bone, and MCP-3 administration partially rescued RANKL-induced bone loss (Fig. 6a–c). These results indicate that in vivo, MCP-3 increases bone mass by inducing bone formation while inhibiting bone resorption.

### Irisin regulates osteoblast and osteoclast differentiation partly via MCP-3 upregulation

**Fig. 7:**
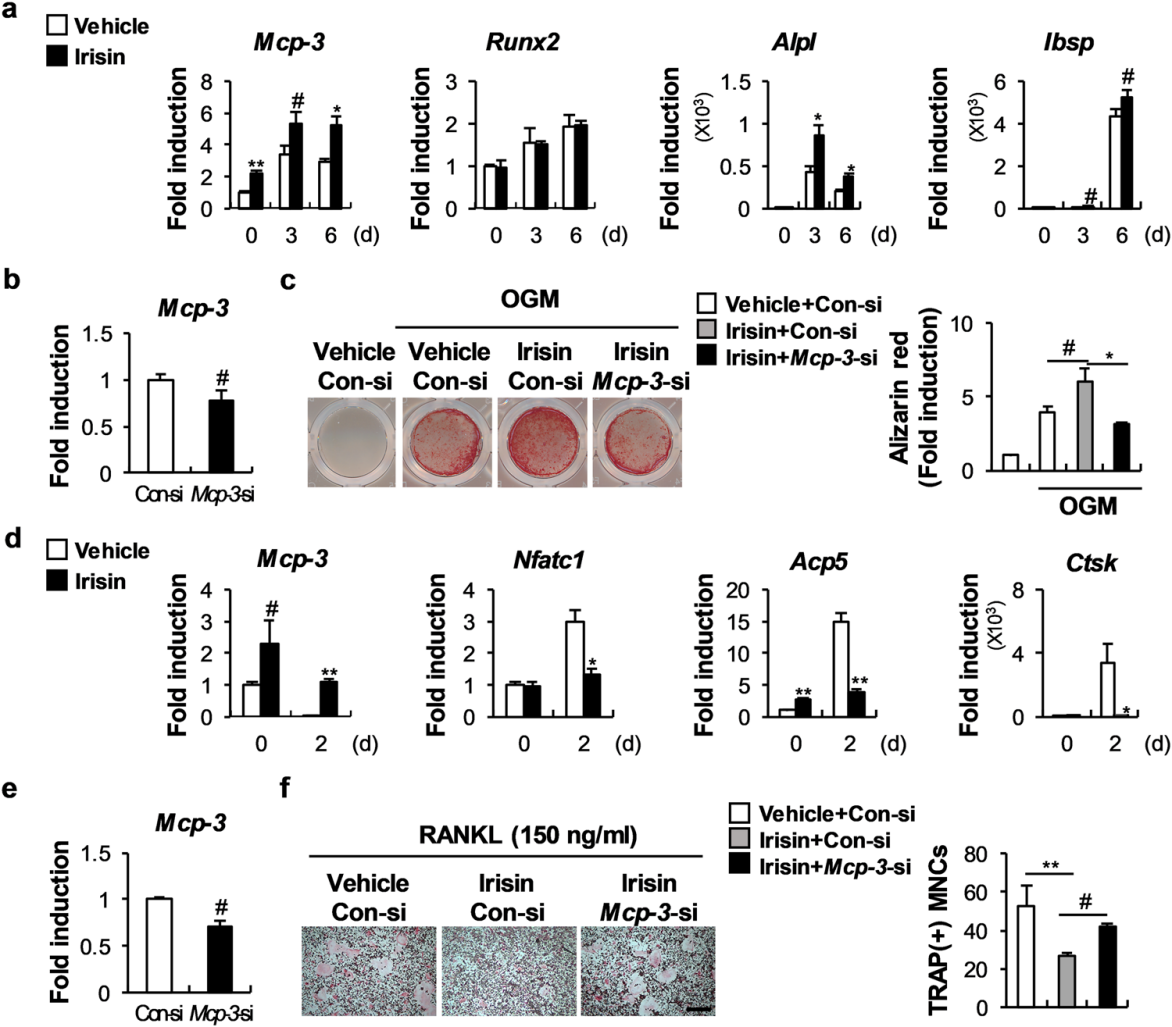
MCP-3 is involved in irisin-regulated osteoblast and osteoclast differentiation. **a** Osteoblasts were cultured in OGM with or without irisin. The relative mRNA levels of the indicated genes were determined via RT‒qPCR (*n* = 3). **b** Osteoblasts were transfected with Con-siRNA or *Mcp-3*-siRNA. The relative *Mcp-3* mRNA level was determined via RT‒qPCR (*n* = 3). **c** Osteoblasts were transfected with Con-siRNA or *Mcp-3*-siRNA and cultured with OGM, with or without irisin. Cultured cells were stained with Alizarin Red and quantified via extraction (*n* = 3). **d** BMMs were differentiated with M-CSF and RANKL, with or without irisin. The relative mRNA levels of the indicated genes were determined via RT‒qPCR (*n* = 3). **e** BMMs were transfected with Con-siRNA or *Mcp-3*-siRNA and then cultured with M-CSF. The relative *Mcp-3* mRNA level was determined via RT‒qPCR (*n* = 3). **f** BMMs were transfected with Con-siRNA or *Mcp-3*-siRNA and cultured with M-CSF and RANKL, with or without irisin. Cultured cells were stained with TRAP, and TRAP-positive cells were counted (*n* = 3; scale bar: 200 µm). The data are presented as the means ± SDs of triplicate samples. #, *, and ** indicate *p* < 0.05, < 0.01, and < 0.001, respectively, vs. the control.

Irisin, a myokine whose expression increases following exercise, inhibits osteoclast differentiation and promotes osteoblast differentiation^22^. Because irisin promotes C2C12 myoblast proliferation by upregulating MCP-3, we investigated whether it stimulates osteoblast differentiation and inhibits osteoclast differentiation by regulating MCP-3 expression^23^. This analysis revealed that during osteoblast differentiation, irisin increased MCP-3 expression and that *Mcp-3* downregulation suppressed irisin-driven nodule formation (Fig. 7a–c). During osteoclast differentiation, irisin upregulated *Mcp-3*, whereas *Mcp-3* downregulation rescued irisin-inhibited osteoclast formation (Fig. 7d–f). Together, these results suggest that MCP-3 partly contributes to the effects of irisin on bone homeostasis by regulating osteoblast and osteoclast differentiation.

## Discussion

Several CC chemokine subfamily members, including CCL2, CCL3, CCL5, and CCL20, have been shown to influence bone remodeling under physiological and pathological conditions^21^. However, the role of MCP-3, a CC chemokine, during bone remodeling is largely unknown.

In this study, in vitro and in vivo analyses revealed that MCP-3 contributes to increased bone mass via the promotion of osteoblast differentiation and the inhibition of osteoclast differentiation. In vitro, recombinant MCP-3 promoted osteoblast differentiation and function, and in vivo, it increased BMP2-induced ectopic bone formation. In vitro, osteoblast-specific MCP-3 overexpression stimulated osteogenic differentiation in calvarial osteoblast precursor cells and BMSCs, and in vivo, it increased bone mass via increased osteoblast number and bone formation. Moreover, in vitro, osteoclast differentiation was strongly inhibited by recombinant MCP-3 and MCP-3 overexpression in osteoclast precursor cells, whereas in vivo, recombinant MCP-3 partially restored RANKL-induced bone loss. In vivo, despite increased MCP-3 secretion, osteoblast-specific MCP-3 overexpression weakly but not significantly suppressed osteoclast numbers (Supplementary Fig. 5). This finding is probably because, in the absence of an autocrine effect, the MCP-3 levels secreted by osteoblasts are not sufficient to inhibit osteoclast differentiation in a paracrine manner. Nevertheless, in vitro and in vivo analyses collectively revealed that MCP-3 increases bone mass by regulating osteoblast differentiation and osteoclast differentiation.

Our data show that in osteoblasts, Ccr3 is a functional MCP-3 receptor. The phosphorylation of p38 by MCP-3 was associated with MCP-3-driven osteoblast differentiation, and these effects (p38 phosphorylation and osteoblast differentiation) were suppressed upon *Ccr3* downregulation, indicating that MCP-3 promotes osteoblast differentiation via Ccr3-dependent p38 phosphorylation. Previous findings indicate that in MLO-Y4 osteocyte-like cells, MCP-3 activates the β-catenin pathway by increasing GSK-3α and GSK-3β phosphorylation^12^. Given that β-catenin pathway activation is important for osteoblast differentiation, MCP-3 might regulate osteoblast differentiation via β-catenin pathway activation. We also showed that MCP-3 affects osteoclasts through Ccr3. In osteoclast precursor cells, MCP-3 attenuated osteoclast differentiation via IFNβ induction, and IFNβ is known to inhibit c-Fos expression^26^. MCP-3 inhibited c-Fos expression (Fig. 3); therefore, MCP-3 likely inhibited osteoclast differentiation through IFNβ-mediated c-Fos inhibition. During osteoclast differentiation, *Ccr3* downregulation in osteoclast precursor cells increased *c-fos* expression but suppressed *Ifnb* expression, restoring the MCP-3-mediated inhibition of osteoclastogenesis. The observation that MCP-3 suppressed RANKL-mediated IκB degradation and JNK phosphorylation independent of Ccr3 suggests that MCP-3 requires other receptors in addition to Ccr3 to inhibit osteoclast differentiation. These observations indicate that MCP-3 induces IFNβ in a Ccr3-dependent manner, while it inhibits RANKL-induced IκB degradation and JNK phosphorylation independent of Ccr3.

A recent study reported that mice deficient in the CC chemokine *Ccl5*, which shares Ccr3 with MCP-3, have impaired bone formation and increased osteoclastogenesis^4^. Together, these findings show that Ccr3 ligands can promote bone formation and inhibit bone resorption, indicating that Ccr3 signaling might stimulate an increase in bone mass. Indeed, adult male Ccr3-deficient mice have a bone phenotype characterized by reduced cortical thickness and volume, which are associated with increased osteoclast activity^20^. However, Ccr3 deficiency was associated with an increase in endocortical osteoid mineralization and greater trabecular and cortical bone formation in mouse long bones^20^. This bone phenotype indicates that Ccr3 deficiency promotes bone formation in vivo. However, our results show that Ccr3 positively regulates bone formation in osteoblasts. The complex chemokine system, whereby specific receptors bind different ligands and specific chemokines or receptor deficiencies are potentially compensated for by other chemokines or receptors, may explain the unexpected finding that Ccr3 deficiency increases bone formation in vivo.

Because irisin, a myokine released during physical activity by skeletal muscles, can improve bone density, it can offer insights into the associations between bones and muscles and the role of exercise in bone health^22,27^. Here, we show that during osteoblast and osteoclast differentiation, irisin upregulates MCP-3 expression and that its effects on both bone cell types are partially suppressed upon the inhibition of MCP-3 expression. These results confirm that MCP-3 has positive and negative roles in osteoblast differentiation and osteoclast differentiation, respectively, and suggest that MCP-3 upregulation is required for the irisin-mediated regulation of bone cell differentiation. In particular, mimicking the effects of exercise by treating C2C12 myoblast cells with AICAR markedly elevated MCP-3 expression (Supplementary Fig. 6), suggesting that, like irisin, exercise may increase MCP-3 levels in skeletal muscles. Therefore, further investigation is needed to determine whether MCP-3 is a myokine that is dependent on or independent of irisin and is increased by exercise, thereby playing an important role as a link between muscle function and bone health.

In conclusion, this study highlights MCP-3 as a potent factor that contributes to increased bone mass by promoting bone formation while simultaneously inhibiting bone resorption. The effects of MCP-3 on osteoblasts and osteoclasts are mediated through Ccr3, but this molecule may regulate the differentiation of bone cell types through other receptors. Our findings indicate that MCP-3 might have therapeutic value against bone loss pathologies, such as osteoporosis.

## Supplementary information

Supplementary Information

## Data Availability

The data used in this study are available from the corresponding author upon reasonable request.
