# Supplementary material for: The MCP-3/Ccr3 axis contributes to increased bone mass by affecting osteoblast and osteoclast differentiation
Source: Exp Mol Med. 2024 Nov 1;56(11):2465–74. doi: 10.1038/s12276-024-01344-6 (PMC11612511; doi:10.1038/s12276-024-01344-6)
Supplement: Supplementary file 1 — Supplementary Information [file 12276_2024_1344_MOESM1_ESM.pdf]

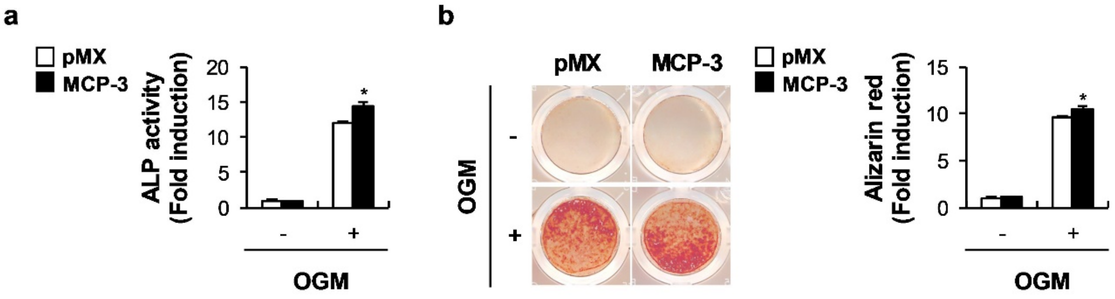


**Supplementary Fig. 1. MCP-3 overexpression in osteoblast precursor cells enhances osteoblast differentiation and function.** (a–b) Osteoblasts were transduced with pMX-FIG (control) or pMX-MCP-3 and cultured in OGM. (a) ALP activity was measured (N = 3). (b) Cells were stained with Alizarin Red and quantified via extraction (N = 3). Data are presented as mean ± SD of triplicate samples. * indicates $p < 0.01$ vs. the control.

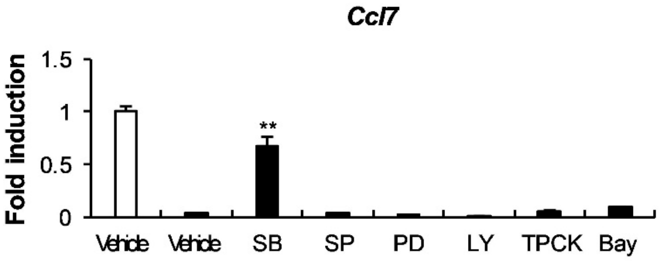


**Supplementary Fig. 2. RANKL suppresses *Mcp-3* expression via p38 phosphorylation.** Osteoclast precursor cells, which were cultured in the presence of M-CSF and RANKL, were treated with various inhibitors, including SB203580 (20 µM), SP600125 (10 µM), PD98059 (20 µM), LY294002 (10 µM), TPCK (5 µM), or Bay (5 µM) for two days. Relative *Mcp-3* mRNA level was determined using RT-qPCR (N = 3). Data are presented as mean ± S.D. ** indicates $p < 0.001$, versus the control.

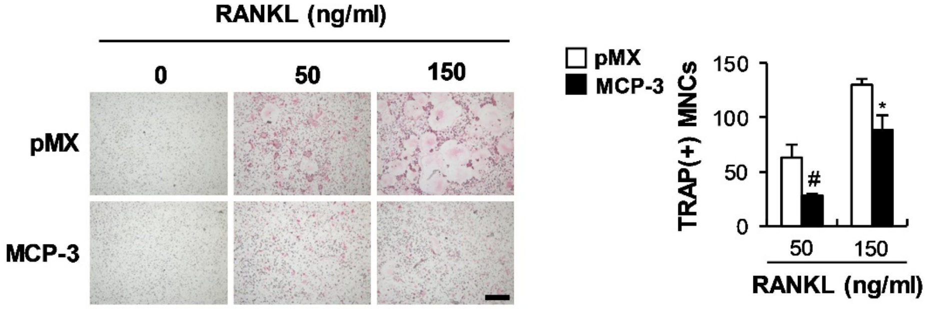


**Supplementary Fig. 3. MCP-3 overexpression in osteoclast precursor cells inhibits osteoclast formation.** BMMs were transduced with pMX-FIG (control) or pMX-MCP-3 and cultured with M-CSF and RANKL. Cultured cells were stained with TRAP, and TRAP-positive cells were counted (N = 3, scale bar: 200 µm). Data are presented as mean ± SD of triplicate samples. #, and * indicate $p < 0.05$ and $< 0.01$, respectively, vs. the control.

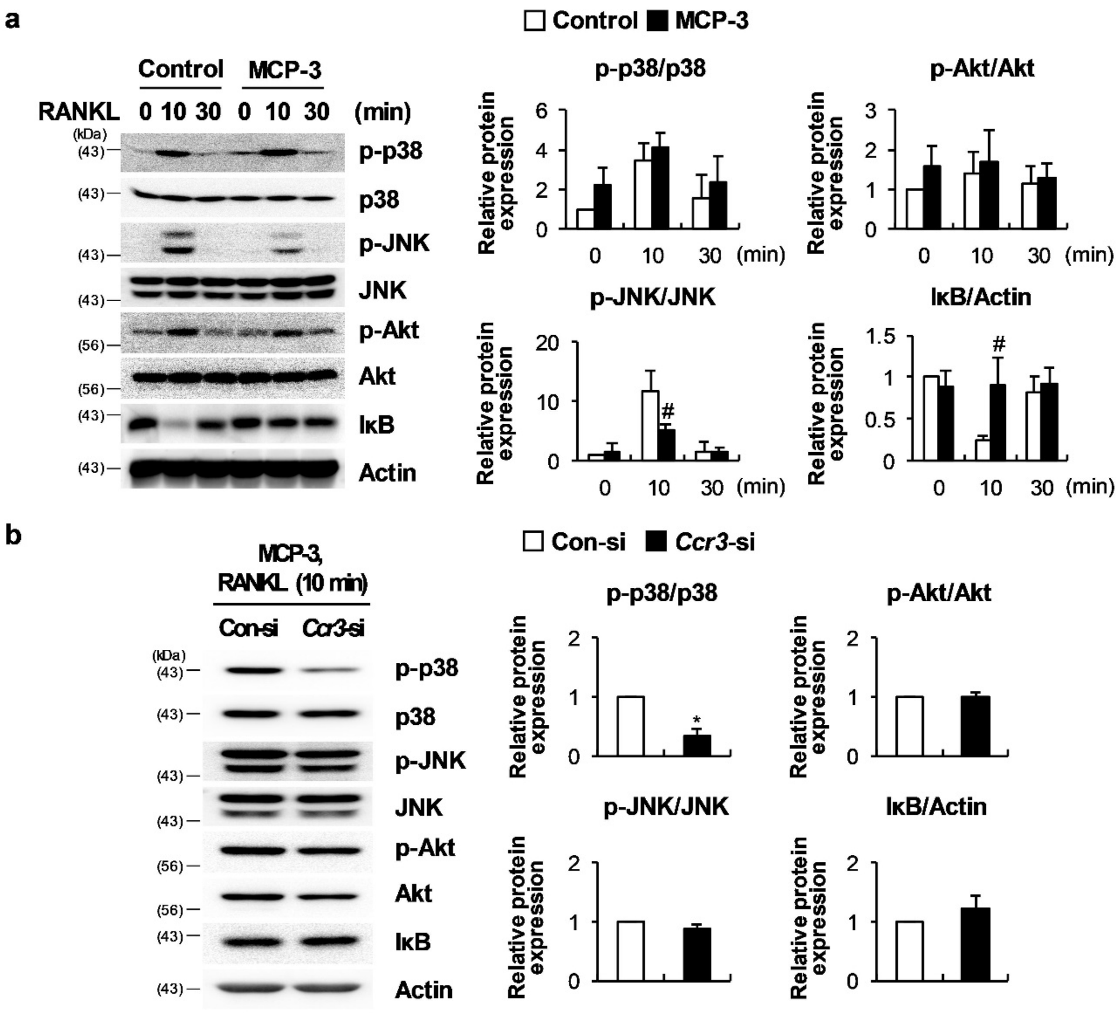


**Supplementary Fig. 4. MCP-3 inhibits RANKL-induced JNK phosphorylation and IκB degradation independently of Ccr3.** (a) Serum-starved BMMs were pretreated with MCP-3, stimulated with RANKL for the indicated durations, and then subjected to western blot analysis of the indicated proteins (N =3). (b) After transfection with Con-siRNA or *Ccr3*-siRNA, BMMs were serum-starved. Cells were pretreated with MCP-3, stimulated with RANKL, and then subjected to western blot analysis of the indicated proteins (N =3). # and * indicate $p < 0.05$ and $< 0.01$, respectively, vs. the control.

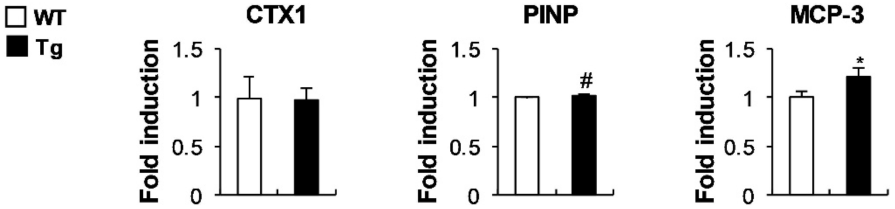


**Supplementary Fig. 5. Transgenic mice overexpressing osteoblast-specific MCP-3 exhibit elevated serum MCP-3 levels.** ELISA was used to determine the levels of CTX1, PINP, and MCP-3 in serum samples from MCP-3 transgenic mice or wild-type littermates (N = 4). Data are presented as mean ± SD of triplicate samples. # and * indicate $p < 0.05$ and $< 0.01$, respectively, vs. the control.

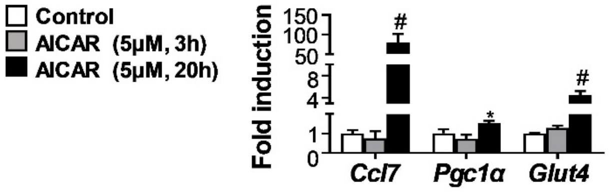


**Supplementary Fig. 6. MCP-3 was elevated in exercise-like cell models.** C2C12 cells were cultured with or without AICAR for the indicated times. The relative mRNA levels of the indicated genes were determined using RT-qPCR (N = 3). Data are presented as mean ± SD of triplicate samples. # and * indicate $p < 0.05$ and $< 0.01$, respectively, vs. the control.
